# Adsorption Kinetics of Polystyrene and Poly(9-anthracenyl methyl methacrylate) onto SiO_2_ Surface Measured by Chip Nano-Calorimetry

**DOI:** 10.3390/polym14030605

**Published:** 2022-02-03

**Authors:** Mina Ishihara, Tomoya Watanabe, Takashi Sasaki

**Affiliations:** Department of Materials Science and Engineering, University of Fukui, Fukui 9108507, Japan; ishiharamina827@gmail.com (M.I.); tenmist1078@icloud.com (T.W.)

**Keywords:** AC chip nano-calorimetry, poly(9-anthracenyl methyl methacrylate), SiO_2_ surface

## Abstract

The alternating current (AC) chip nano-calorimetry is a powerful tool to investigate the physical properties of polymer thin films. In this paper, we report on the adsorption kinetics of polymers in which an AC chip nano-calorimetry was used for the first time. This technique allows for the real-time measurement of the adsorption kinetics of polymer chains onto the SiO_2_ surface. We used polystyrene (PS) and poly(9-anthracenyl methyl methacrylate) (PAMMA), which have different chemical natures and side group sizes. It was confirmed that the observed adsorption kinetics for PS were consistent with previously reported results obtained by dielectric spectroscopy. For PAMMA, we found characteristic adsorption kinetics, which shows a clear kink at the crossover between the early and later stages, while PS exhibits a lesser tendency of showing the kink as demonstrated by previously reported results.

## 1. Introduction

Polymer thin films are promising materials that facilitate advances in the industry and improvements in our daily lives. Due of their large surface-to-volume ratio in confined geometries, their properties, such as gas permittivity, density, creep behavior, relaxation time, and physical aging, significantly deviate from the bulk properties [1,2,3,4,5,6,7]. Additionally, the characterization of atomic/molecular interactions at the interface between polymeric and inorganic materials is becoming of more importance not only academically but also practically. For example, Menazea et al. extensively studied the interaction between lithium titanate and polymer blends to improve their performance as electrode materials for batteries [8]. To tailor the properties of polymer/inorganic composite materials, it is necessary to understand the behavior of the polymer chains near the interface. Recently, the adsorbed polymer layers have gained significant attention because a correlation was identified between the number of adsorbed chains and material properties [9,10,11,12].

The procedure of “Guiselin’s experiment” provides the adsorbed layer by annealing and subsequent solvent washing [13]. Many experiments have shown that the residual adsorbed layer thickness depends on the annealing time at an elevated temperature [14,15,16,17,18,19]. Dielectric spectroscopy provided real-time measurements of adsorption kinetics in a buried interface with an aluminum substrate [20]. This real-time measurement provides important information because the adsorption kinetics can be revealed without solvent washing.

Housmans et al. proposed a description of the kinetics of the irreversible adsorption, including a crossover between the linear and logarithmic growth stages [21]. Many experiments have revealed linear and logarithmic adsorption kinetics, and to the best of our knowledge, this assumption has been confirmed only by studies using Guiselin’s experiment [21,22,23,24]. This two-stage mechanism is consistent with the bimodal growth reported for the chains adsorbed from solutions [25]. However, Roth et al. suggested an open question as to what the difference in solubility is of the layer adsorbed from melt and from the solution [16]. As already pointed out by Granick in 2002, the relationship between the loop size and the dynamics of the entire polymer film is still unknown [26]. Jiang et al. proposed that the magnitude of the polymer/substrate interactions controls not only the final thickness, but also the kinetics of the flattened adsorbed layer formation [24]. However, this can only be explained qualitatively by simulation results: the number of polymer chains adsorbed on solids increases with an increase in the solid-segment interaction. In summary, because of its intricacy, the adsorption mechanism is not completely understood.

The alternating current (AC) chip nano-calorimetry is a technique that can detect the heat capacity of a sample of the order of several nanograms [27]. Similar to the dielectric relaxation measurements, an embedded measurement that measures the heat capacity of the entire film can be performed, so that the adsorption experiment can be measured in real time. Studies on the glass transition temperature of the polymer thin films have been conducted via AC chip nano-calorimetry [3,27,28,29,30,31]. However, there is no record of this technique being applied to the direct measurement of the polymer adsorption process. The surface of the chip sensor used for the calorimetry was coated with SiO_2_. Dynamic measurements can be performed on the SiO_2_ substrates that cannot be used by the dielectric relaxation measurements, where the substrate needs to have conductivity. In the future, this advantage may enable the study of adsorption of modified substrates by surface-fluorination. In this study, the AC chip nano-calorimetry is used to investigate the adsorption kinetics.

In this study, we establish a technique for observing the formation process of an adsorption layer via AC chip nano-calorimetry for the first time. Using this technique, we measure the time evolution of the heat capacity of polymers during adsorption onto a silica substrate, which directly reflects the adsorption kinetics. We use polystyrene (PS) because many studies have been conducted on this polymer in a previous work [18]. We also use poly(9-anthracenyl methyl methacrylate) (PAMMA), which is quite characteristic of its bulky anthracenyl group. Comparing the results of these polymers, we attempt to reveal the influence of the bulky side chains on the adsorption process. In addition, the anthracenyl group exhibits strong absorbance in ultraviolet (UV) range, thus PAMMA is useful for measuring the film thickness via UV-vis spectroscopy.

## 2. Materials and Methods

### 2.1. Materials

**Figure 1:**
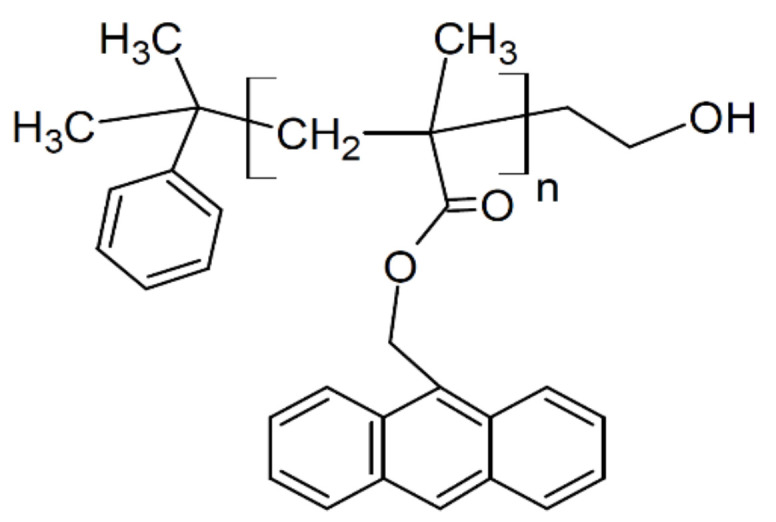
Chemical structure of PAMMA.

The atactic polystyrene (PS, *M*_n_ = 424 kDa; *M*_W_/*M*_n_ = 1.06) was purchased from Scientific Polymer Products (Ontario, NY, USA). PAMMA (*M*_n_ = 88.0 kDa; *M*_W_/*M*_n_ = 1.15 was purchased from Polymer Source, Solon, OH, USA (see Figure 1). The polymers were used as received without further purification. The glass transition temperatures *T*_g_ for PS and PAMMA were measured to be 379 K and 418 K, respectively, via differential scanning calorimetry (DTG-60, Shimadzu, Kyoto, Japan) on the second heating at 20 °C/min under a nitrogen atmosphere. The molar extinction coefficient *ε* of the anthracenyl groups in PAMMA was determined to be 6713 M^−1^ cm^−1^ at 370 nm via UV-vis spectroscopy using a Hitachi (Tokyo, Japan) U-3900H. Toluene was obtained from Kanto Chemicals, Tokyo, Japan. Chloroform was obtained from Fujifilm Wako Pure Chemicals, Tokyo, Japan, and used as a good solvent for PAMMA.

### 2.2. Heat Capacity Measurement

A spin-coated PS film (approximately 88 nm in thickness) was prepared from a 1.0 wt% toluene solution at 1000 rpm for 30 s. The film thickness of PS was estimated using an optical interferometer, Filmetrics (Yokohama, Japan) F20. The film was then cut into pieces of approximately 2 mm × 2 mm in size and then floated on water. One of the pieces was scooped up with a chip sensor and placed in the active area. Then, it was dried under vacuum at room temperature for 24 h.

A PAMMA film was cast directly onto the chip sensor from a saturated chloroform solution (0.26 wt%). The thickness of the films was 1.0~1.2 μm, which was controlled by the amount of the solution. We confirmed that the cast film was spread over the entire sensor area of 7.24 mm^2^. The thickness of the cast film *h* was estimated as follows:

(1) $$h=\frac{x\rho_{\mathrm{sol}}\varphi }{\rho_{\mathrm{poly}}A}$$

where x is the volume of the drop, $\rho_{\mathrm{sol}}$ is the density of the solvent, $\rho_{\mathrm{poly}}$ is the density of the polymer, *φ* is the concentration of the PAMMA/chloroform, and *A* is the surface area of the active area of the chip sensor. After casting the PAMMA film, the samples were dried in a vacuum oven at room temperature for 24 h. The heat capacity of the entire PAMMA film during adsorption at 433 K (*T*_g_ + 15 K) was measured via heat capacity measurements.

**Figure 2:**
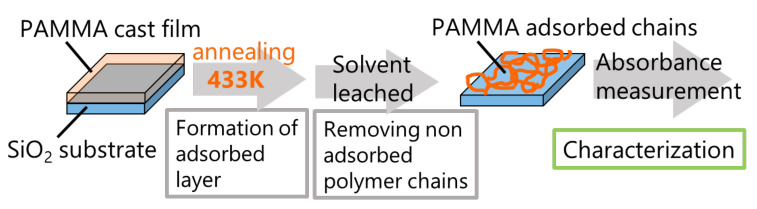
Preparation of PAMMA adsorbed layer via the Guiselin approach.

We followed the established protocol using the Guiselin approach [32,33,34,35] for PAMMA-adsorbed layers (Figure 2). Optically polished SiO_2_ (2 cm × 2 cm) substrates were cleaned using the following procedure. First, the SiO_2_ substrates were heated at 973 K for 10 min to remove organics in an electric furnace, and then, these substrates were rinsed with methanol. PAMMA films of 800 nm thickness were cast from a saturated solution of 100 μL onto cleaned SiO_2_ substrates, and then left at room temperature for 10 min. Subsequently, the samples were annealed at 433 K in a vacuum oven for various annealing times (*t*_ads_). The samples were then solvent leached in a bath of chloroform at 40 °C for 1 h and then rinsed with fresh chloroform to remove the non-adsorbed polymer chains. This washing procedure was repeated three times. The amount of residual adsorbed layer was evaluated via UV-vis spectroscopy.

### 2.3. Sample Preparation

**Figure 3:**
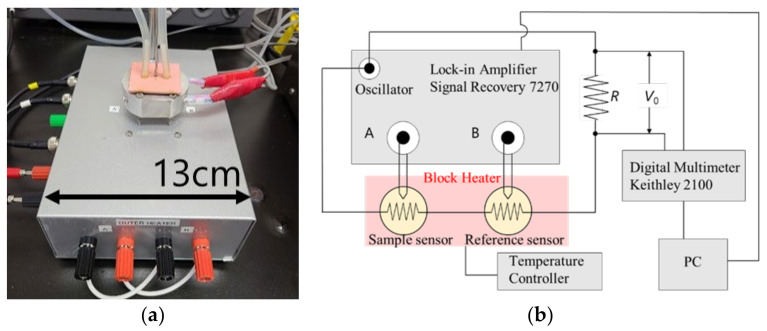
Equipment of AC chip nano-calorimetry. The two chip sensors are included in the aluminum block heater (**a**); (**b**) shows the schematic picture of the electric setup.

Heat capacity measurements were performed using an AC chip nano-calorimeter (XI-39390, Xensor Integration, Delfgauw, Netherland). The equipment used for the AC chip nano-calorimetry is shown in Figure 3. Two chip sensors were used for the sample and the reference (empty). The set temperature was adjusted using a handmade aluminum block heater that covered the two chip sensors. The measurements were performed under a nitrogen flow of 0.1 mL/min.

The amplitude difference Δ*V* = |*V*_A_ − *V*_B_| and the phase difference *θ* between the sample and the reference were obtained, where *V*_A_ and *V*_B_ are the voltages from the thermopile of the sample and the reference, respectively. In AC calorimetry, the apparent heat capacity is given by [30].

*C_p_* = Δ*V aiωC*_0_^2^/*P* (2)

where *a* is the reciprocal of the sensitivity of the thermopile, which was estimated to be 452 K V^−1^; *C*_0_ is the effective heat capacity of the empty chip; and *P* is the heating power applied to the chip sensors, which can be evaluated from the voltage *V*_0_. The voltage *V*_0_ over the known resistor *R* (shown in Figure 3) was measured using a digital multimeter Keithley 2100 (Solon, Quebec, Canada). The chip sensors in the block heater were driven by an alternating current at a frequency of *ω*/2 from the oscillator of a lock-in amplifier Signal Recovery (Ametek, Berwyn, PA, USA) 7270. We also obtained the data of the phase angle difference *θ* between the sample and the empty sensors. The real part of the complex heat capacity is approximately proportional to Δ*V* cos *θ*. While measuring the change in the complex heat capacity during polymer adsorption, the difference in the temperature amplitude between the sample chip (chip A) and the reference chip (chip B) connected in series was observed. The temperature amplitude is inversely proportional to the heat capacity of the sample. When adsorption occurs, the heat capacity of the sample decreases, the temperature amplitude increases, and the absolute value Δ*V* decreases. Therefore, it is expected that the above measured value (i.e., the difference in the voltage amplitude between chips A and B) will decrease as the adsorption proceeds.

We attempted to observe the dynamics of the polymer chains near the chip interface by measuring the heat capacity. The storage heat capacity (*C*’(*t*)) at a temperature modulation of 200 Hz was measured at 429 K for a chip sensor on which a PS spin-cast film was placed. Nitrogen was infused at 1 mL/min for 10 min prior to the measurement. At the same time, the temperature of the block heater was set such that the temperature of the sample became *T*_g_ + 50 K. It took approximately 10 min to reach the set temperature. Then, the flow rate of nitrogen was set to 0.1 mL/min, and after 1 min had passed, the measurement was started. PAMMA was also investigated at 433 K (*T*_g_ + 15 K) in the same manner as that for PS.

### 2.4. Thickness Determination of the Adsorbed Layer

The amount of PAMMA adsorbed was investigated using a UV-vis spectrophotometer. Since the anthracenyl group showed the maximum absorbance at a wavelength of 370 nm, the absorbance was measured in the wavelength range of 300–450 nm. The absorbance was converted to the thickness of the adsorbed layer using the Lambert–Beer law as follows:

*A* = *εcl* (3)

In this equation, *A* is the absorbance, *ε* is the molar extinction coefficient of the anthracenyl group (6713 M^−1^ cm^−1^), and *c* is the concentration of the anthracenyl group per volume of PAMMA (4.27 mol L^−1^). The optical path length *l* was assumed to be the thickness of the adsorbed layer. The concentration of the anthracenyl group was determined by dividing the density of PAMMA (1.21 g cm^−3^) by the molecular weight per repeating unit (276.3 g mol^−1^).

## 3. Results and Discussion

It was confirmed that the polymer films prepared in this study were uniform as evidenced by the optical microscopy before the adsorption. However, we found that cracking occurred occasionally for PAMMA films after the long-time annealing, which was probably caused due to dewetting during the adsorption process. Typical images of the annealed samples with cracking are shown in Figure S1 in the Supplementary Materials. Multiple experiments repeated carefully revealed that such cracks did not directly affect the heat capacity profile obtained by the nano-calorimetry. This suggests that the crack did not reach the region of the adsorption layer near the interface. On the other hand, no cracks were observed for PS films even after the annealing.

**Figure 4:**
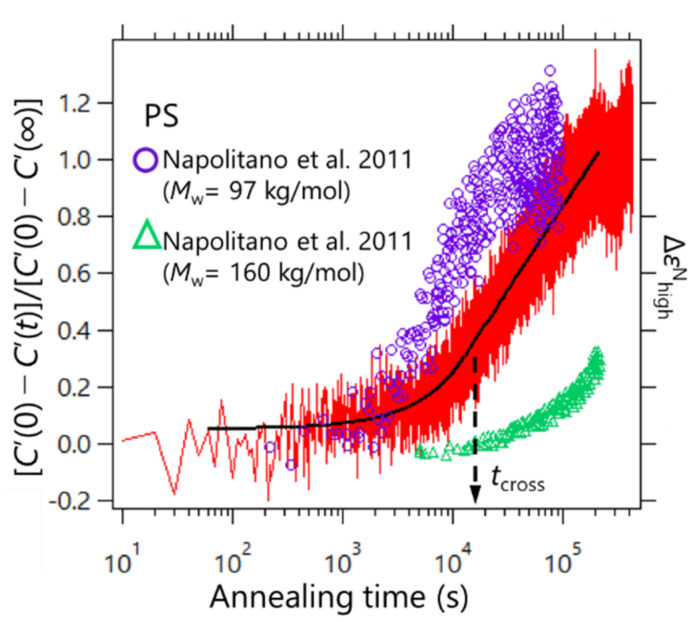
Time evolution of the normalized storage heat capacity of PS (*M*_w_ = 424 kDa) layer at 429 K (red line, left axis). The purple circles and green triangles show the literature data of dielectric measurements for PS on Al_2_O_3_ substrates at 423 K with *M*_w_ = 97 kDa and 160 kDa, respectively (reproduced from Ref. [20] Copyright Springer Nature (2011)) (right axis). The black solid line indicates the fitting results with Equation (4).

*C*’(*t*) was normalized so that it started from zero to the final value of unity as [*C*’(0) − *C*’(*t*)]/[*C*’(0) − *C*’(∞)]. To determine the adsorption kinetics of PS, the heat capacity measurements were performed via the AC chip nano-calorimeter at 429 K. The results are shown in Figure 4. For comparison, the results of the adsorption kinetics data for PS obtained by Napolitano et al. via the dielectric spectroscopy are also plotted in Figure 4 [20]. The data were obtained for two different molecular weights of 97 and 160 kDa at 423 K (purple circles and green triangles, respectively). The molecular weight of PS used in this study was 424 kDa, which showed an intermediate time evolution profile between those obtained from the dielectric measurements. This may have resulted from the difference in the chemical structure of the substrate; the SiO_2_ surface was used in our experiment, and Al_2_O_3_ was used in the dielectric spectroscopy reported by Napolitano et al. These differences in the chemical structure of the substrate may affect the adsorption kinetics. The effect of polymer/substrate interaction on the polymer adsorption kinetics has been discussed by other groups [24,34,35]. It has been revealed that the adsorption rate is reduced when the polymer/substrate interaction becomes strong. Additionally, when porous substrates were used, the adsorption is also slowed as the size of the pore becomes smaller. The latter suggests that the confinement of polymer chains in the pores also affects the adsorption kinetics.

Furthermore, the observed adsorption kinetics were in line with the two regimes proposed in Ref. 21. Housmans et al. revealed the growth kinetics of heterogeneous structures in the vicinity of the polymer/substrate interface, which can be described by [21].

(4) $$h_{ads}=\left\{\begin{array}{c} h_{0}+vt\\ h_{cross}+\Pi \log t \end{array}\right.$$

where *h*_0_ is the thickness of the adsorbed layer at *t* = 0; *v* and *Π* express the growth rates in the first and second regimes, respectively; *h*_cross_ is the value of the thickness at the crossover time *t*_cross_; and the value of *t* in the argument of the logarithm is normalized by *t*_0_ = 1 s, to ensure correct dimensionality. This means that, for a short time, the growth kinetics follows a linear time dependence where the adsorbed layer grows by the direct pinning of segments to the substrate, and the logarithmic regime was reached where the diffusion of segments through the already existing layer with entropy was lost. We estimated the crossover time via nonlinear least squares fitting processes with Equation (4) for the two temporal regions. As a result, *t*_cross_ was estimated to be ca. 1.5 × 10^4^ s, as shown in Figure 4. Furthermore, our data do not exhibit a clear kink at *t*_cross_, but similar profiles were confirmed for the dielectric relaxation measurements by Napolitano et al. Such an unclear kink between the two regimes for PS may be due to a dynamic measurement feature.

**Figure 5:**
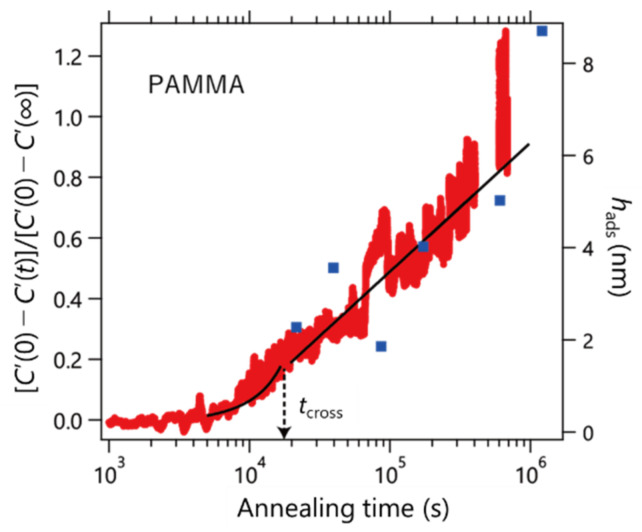
Time evolution of the normalized storage heat capacity of the PAMMA (*M*_w_ = 88.0 kDa) layer at 433K (left axis). The solid line indicates the fitting results with Equation (4). Blue squares are the thickness of the adsorbed layer prepared using the Guiselin approach (right axis).

We extended our experiments to PAMMA carrying an anthracenyl group (bulky side chain). Figure 5 shows the results. As a result of fitting Equation (4), *t*_cross_ was evaluated to be 1.9 × 10^4^ s. The right axis shows the thickness of the adsorbed layer prepared using the Guiselin approach (blue square). From these results, it was found that the heat capacity and thickness of the adsorption layer changed consistently with the annealing time. It was found that the boundary between the linear and logarithmic profiles was not smooth compared to the case of PS. This result means that the rates of change of *v* and *Π* are different, as shown in Table 1. The difference in the adsorption behavior between PS and PAMMA observed in this study evokes the assumption suggested by Gin et al. that the local conformations (i.e., trans and gauche states) of a polymer chain play a vital role in the unusual densification process during adsorption [14]. The correlation between the adsorption kinetics and chemical structure of the side chain should be elucidated. The data in Figure 5 revealed an induction period before the adsorption commences. This results in an apparent negative value of *h*_0_ as shown in Table 1, but of course, it is not the real value of the initial thickness of the adsorbed layer. The bulky anthracenyl group might require such an induction period in which slow conformational rearrangements take place to achieve favorable structures for the adsorption process at the interface.

## 4. Conclusions

We investigated, for the first time, the adsorption process for polymers via AC chip nano-calorimetry. The main conclusions are summarized as follows: (1) we were able to measure the adsorption of polymers on a silica substrate using the nano-calorimetry technique. Such real-time measurements can provide reliable information about the adsorption kinetics of polymers on a silica substrate. (2) The obtained profile of the adsorption kinetics for PS was consistent with those obtained via the dielectric spectroscopy reported in the literature. Both the adsorption kinetics of PS from the AC chip nano-calorimetry and dielectric spectroscopy show an unclear transition between the earlier and later regimes at the crossover time, which may be a feature of the dynamic measurement. (3) On the other hand, PAMMA that has bulky side groups clearly exhibits a different adsorption profile from PS. The fitting parameter of PAMMA shows a clear crossover between the two regimes compared with that of PS.

The results obtained in this study provide typical evidence that the chemical structure of the polymer influences significantly its adsorption behaviors at the interface. Such a valuable finding gives a clue to elucidate the interfacial phenomena based on the chemistry of the materials on a molecular basis, and provides a molecular guideline for the development of adhesion technology and molecular design for various composite materials. We are currently investigating how the relationship between the polymer/substrate interaction and the adsorption process is related to the adsorption rate.

## Figures and Tables

**Table 1:** Obtained parameters from fitting analysis with Equation (4).

Polymer	*h*_0_ (nm)	*v* (nm s^−1^)	*Π*	*Π*/*v*
PS	0.51 ± 0.004	1.98 × 10^−5^	0.584 ± 0.002	2.95 × 10^4^
PAMMA	−0.66 ± 0.002	1.62 × 10^−5^	0.425 ± 0.001	2.62 × 10^4^

## Supplementary Materials

The following are available online at https://www.mdpi.com/article/10.3390/polym14030605/s1. Figure S1. Optical micrographs of typical PAMMA films on the active area of the chip sensor after annealing at 433 K for ca. 10^6^ s. The scale bar indicates 70 μm.
