# Supplementary material for: Adsorption Kinetics of Polystyrene and Poly(9-anthracenyl methyl methacrylate) onto SiO2 Surface Measured by Chip Nano-Calorimetry
Source: Polymers (Basel). 2022 Feb 3;14(3):605. doi: 10.3390/polym14030605 (PMC8839510; doi:10.3390/polym14030605)
Supplement: Supplementary file 1 [file polymers-14-00605-s001.zip › polymers-1558459-supplementary.pdf]

polymers

MDPI

# Supplementary Materials: Adsorption Kinetics of Polystyrene and Poly(9-anthracenyl methyl methacrylate) onto $SiO_2$ Surface Measured by the Chip Nano-Calorimetry

**Mina Ishihara, Tomoya Watanabe and Takashi Sasaki**

**S1. Morphology of the prepared films of PAMMA**

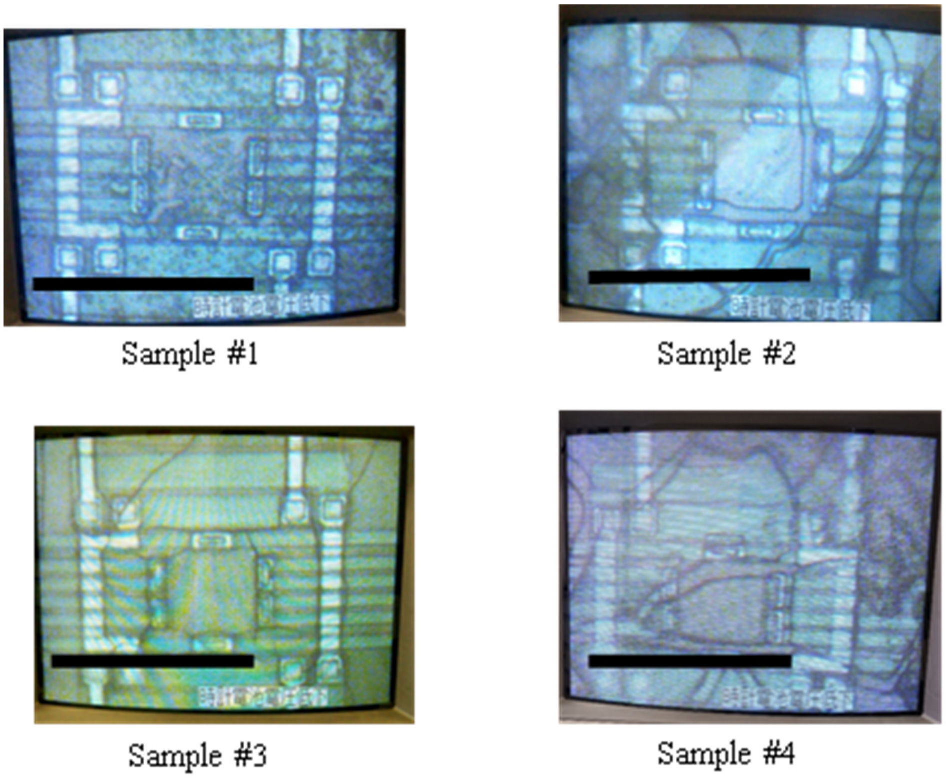


**Figure S1.** Optical micrographs of typical PAMMA films on the active area of the chip sensor after annealing at 433 K for ca. $10^6$ s. The scale bar indicates 70 μm.
